# Vaccine management systems with a focus on cold chain management and delivery of immunization services in low- and middle-income countries: A scoping review

**DOI:** 10.1371/journal.pone.0350231

**Published:** 2026-06-16

**Authors:** Amatullah Sana Qadeer, Nirupama AY, Winnie Paulson, Jennifer Rachel, Lipika Nanda, Ambarish Dutta, Sarit Kumar Rout

**Affiliations:** 1 Indian Institute of Public Health-Hyderabad, PHFI Institute of Public Health Sciences (Deemed to be University), Hyderabad, Telangana, India; 2 Indian Institute of Public Health-Bhubaneswar, PHFI Institute of Public Health Sciences (Deemed to be University), Hyderabad, Telangana, India; Freelance Consultant, Myanmar, MYANMAR

## Abstract

**Objective:**

The main objective of this scoping review is to examine the characteristics of vaccine management and delivery systems with a specific focus on cold chain management used for routine immunization in the LMICs, along with factors influencing these systems, challenges faced and the extent of digitization implemented.

**Methods:**

A comprehensive literature search was conducted using PubMed, Scopus, Cochrane Library, and Google Scholar for studies published between 2010 and February 2025. Our study selection, data charting, and synthesis followed methodological frameworks outlined by the Joanna Briggs Institute and PRISMA guidelines for Scoping Review.

**Result:**

Outof 2451 identified studies, 16 eligible studies were included. The findings revealed diverse vaccine management system designs, including centralized and decentralized architecture, interoperable integrated models, and provisions for online and offline accessibility. Core features of these systems included longitudinal health records, automated inventory monitoring, and beneficiary engagement features including digital health cards. Implementation was influenced by global initiatives, project management teams and their capability, and challenges related to digitization. Factors impacting vaccine delivery included immunization sessions, stock management systems, distribution weaknesss, suboptimal practices, training gaps, and documentation. Digital transformations varied across countries, with Vietnam, Tanzania, and Zambia adopting electronic immunization registries, while India implemented the Electronic Vaccine Intelligence Network. The implementation of these digital logistics management information systems significantly improved vaccine management by reducing wastage, enhancing record-keeping, and optimizing supply chain efficiency.

**Conclusion:**

To our knowledge, this scoping review is the first to provide empirical evidence on vaccine management and delivery systems and digitization in immunization in the LMICs. This review concludes that LMICs have been adapting various systems including electronic system, digitization, decentralisation of vaccine delivery, stock monitoring, individual tracking to improve immunization. Across countries, some have made notable progress, while others are at their initial stage of implementation. However, this review suggests further studies on integrated vaccine management systems and their impact for inter-country comparison.

## 1 Introduction

Globally, around 5 million children die each year due to preventable diseases [1]. Although notable achievement has been observed in the reduction of child mortality, the under-five mortality rates are still high, especially in the developing countries. Immunization is one of the most cost-effective community health interventions for averting infectious diseases and associated deaths [2]. Immunization program is estimated to be preventing over 3 million deaths per year globally [3]. According to the WHO and UNICEF database, currently, 14.3 million infants are not vaccinated in the world. That is 4 million more than the 2024 target needed to stay on track with Immunization Agenda 2030 goals. Data from 195 countries show that 131 countries have consistently reached at least 90% of children with the first dose of DTP vaccine since 2019, but there has been no significant movement in expanding this group. The humanitarian crisis causes slow progress in vaccination in many of the countries. Around a quarter of infants stay in 26 countries affected by conflicts and human crisis and half of these children in these countries are not vaccinated [2,4].

Vaccination plays a critical role in the reduction of child mortality and is a important aspect of health systems strengthening [5]. According to the WHO “vaccination is a simple, safe, and effective way of protecting against harmful diseases, before one comes into contact with them. It uses your body’s natural defences to build resistance to specific infections and makes your immune system stronger [6]. This increases long term productivity, improves education attainment, prevents diseases for the marginalised groups and averts loses due to disability and death [7]. The first step in vaccination is packaging at the level of producers and transporting is to be done appropriately [8]. Vaccines are biopharmaceutical products that require to be maintained at a specific temperature so that their potency is retained [9]. Therefore, it is essential to maintain the required temperature throughout the supply chain until the final delivery point. According to the WHO recommendations, most vaccines need to be kept at a temperature range of +2 and +8 °C [10]. However, this process requires the involvement of multiple stakeholders, logistics of cold chain systems, surveillance methods, and proper delivery of vaccines from inception till the final destination, thereby constituting a complex process with multi-fold challenges [10,11].

The cold chain system depends on reliable equipment- cold room, freezer room and walk-in coolers/freezers, In-Line Refrigerators(ILR) and devices which monitor temperature [10]. The maintenance of the cold chain and monitoring of temperature is a major challenge in developing countries due to lack of reliable cold storage equipment, inadequate knowledge and training of handlers, and improper cold chain management practices. This in turn results in high rates of vaccine wastage and reduced immunization coverage [10,12]. The WHO defines vaccine wastage as “loss by use, decay, erosion, or leakage or through wastefulness”, and can be calculated as the proportion of vaccine administered against vaccine issued [13,14]. Vaccine wastage is categorized into wastage in opened vials, and unopened vials [14]. It can be caused by various reasons such as expiration of vaccine before usage, inconsistent temperature storage or inadequate cold chain management, physical damage, losses in transit or inventory, or incomplete use of the nominal number of doses in multi-dose vials [15]. Vaccine waste measurements vary globally based on the reporting infrastructure of countries, while some countries use real-time digital tracking and others rely on manual logs and projected vaccine estimates [16,17]. These measurements also change based on the national policies such as zero missed opportunities, resulting in higher recorded wastage in rural areas where multi-dose vials are opened for less patients for immediate immunization [14,18]. Therefore, it is essential to maintain a sustainable vaccine supply chain system so that routine immunizations can be carried out without any interruptions and limit the spread of infectious diseases.

The effectiveness of vaccines relies on the infrastructure, equipment, and systems along with processes required to transport them from manufacturing facilities to the remote areas. Without efficient and secure immunization cold chains capable of managing, storing, transporting, and distributing vaccines to everyone, the delivery of immunization and maternal and child health services will not reach their maximum effectiveness [12,19]. Hence, by improving cold chain systems, it is possible to expand the reach of effective immunization coverage and consequently reduce the child mortality rate resulting from vaccine-preventable diseases [20]. In the entire immunization process, vaccine management systems and delivery are crucial and this refers to the overall handling of the vaccines from manufacturer to patients covering logistics, cold chain management system, storage and data. Cold chain management system is one of the important indicators in the vaccine management system which emphasises on maintaining appropriate temperature for vaccine potency. There is lack of evidence on the factors affecting cold chain management system and it’s influences on vaccine delivery system. In this scoping review, we aimed to explore the characteristics of vaccine management system with a specific focus on cold chain management system and delivery of immunization services in the low- and middle-income countries (LMICs). This review will also identify the extent of digitization within these systems, as digital tools have shown potential in improving vaccine stock management, temperature monitoring, and vaccination scheduling. As the degree of digitization varies across countries, and understanding this variation can help find out where digital interventions could be most impactful [21]. Countries included in the review have adopted different systems providing opportunities to others to learn the new practices and innovations. Therefore, this scoping review aims to identify the characteristics of different vaccine management systems for routine immunization in the LMICs, factors affecting these systems, and the extent of digitization adopted to improve vaccine delivery for routine immunization in the LMICS. By conducting this scoping review, we aspire gather comprehensive information on these aspects, which can inform the design of interventions, and improve immunization outcomes in the LMICs.

## 2 Methodology

### 2.1 Methodological approach

We adopted scoping review method for this study as it provides a comprehensive overview while including different types of available evidence and the scoping review seemed better suited for the given objectives. We used a structured and comprehensive approach in this scoping review to identify, map, and synthesize the existing literature on vaccine management and systems in the LMICs. The LMICs were defined by the world bank as countries whose gross national income per capita varies between 1136$ and 4495$ [22]. This review adhered to the methodological framework developed by the Joanna Briggs Institute (JBI) for evidence synthesis, originally developed by Arksey and O’Malley and the Preferred Reporting Items for Systematic reviews and Meta-Analyses Extension for Scoping Reviews (PRISMA-ScR) guidelines [23,24]. A PRISMA-ScR Checklist was used for standardized quality reporting and transparency (Appendix 1).

### 2.2 Selection of sources of evidence

The literature search was conducted on 12^th^ February, 2025 across electronic databases, including PubMed, Scopus, Cochrane Library, and Google Scholar. The search was limited to studies published between 2010 and 12^th^ February 2025. A detailed description of the search strategy, including specific terms searched and conventions applied, can be found in Appendix 2. The search results were imported into the Rayyan software, which facilitated the identification and removal of duplicate records.

### 2.3 Study selection

#### 2.3.1 Inclusion criteria.

To be eligible for inclusion, studies had to meet the following criteria:

1. Studies on vaccine cold chain management system and delivery for routine immunization.
2. Studies exploring electronic vaccine cold chain management.
3. Observational, intervention, operational study designs.
4. Studies in the LMICS.
5. Studies available in English language.

#### 2.3.2 Exclusion criteria.

Studies were excluded if they were:

1. Case report studies and poster abstracts.
2. Studies on vaccines not included in Routine Immunization.
3. Studies published before 2010.

### 2.4. Data charting and management

The study selection process was carried out by a team of three reviewers [ASQ, JR, WP], of which two reviewers [ASQ and WP] screened the titles, abstracts, and full-text articles to determine eligibility using Rayyan software and any disagreements were resolved by the third reviewer [JR]. A standardized data extraction framework was developed to systematically capture key information from the included studies, such as the title, authors, year of publication, study location, study design, population, conceptual focus of the included study, contextual factors that affect vaccine management. This also focused on cold-chain management, features and names of vaccine management and cold chain management systems, and digitization software used. The synthesized data was presented in a narrative format.

The data charting form was developed by two authors [ASQ and SKR] to determine variables for data extraction. Three authors [ASQ, WP, NAY] were responsible for the data extraction and charting process. SKR provided conceptual guidance including the objective and methodology finalisation and finally reviewed the article. Additionally, SKR was involved in writing as well as proof reading of the article at different stages.

## 3 Result

**Fig 1:**
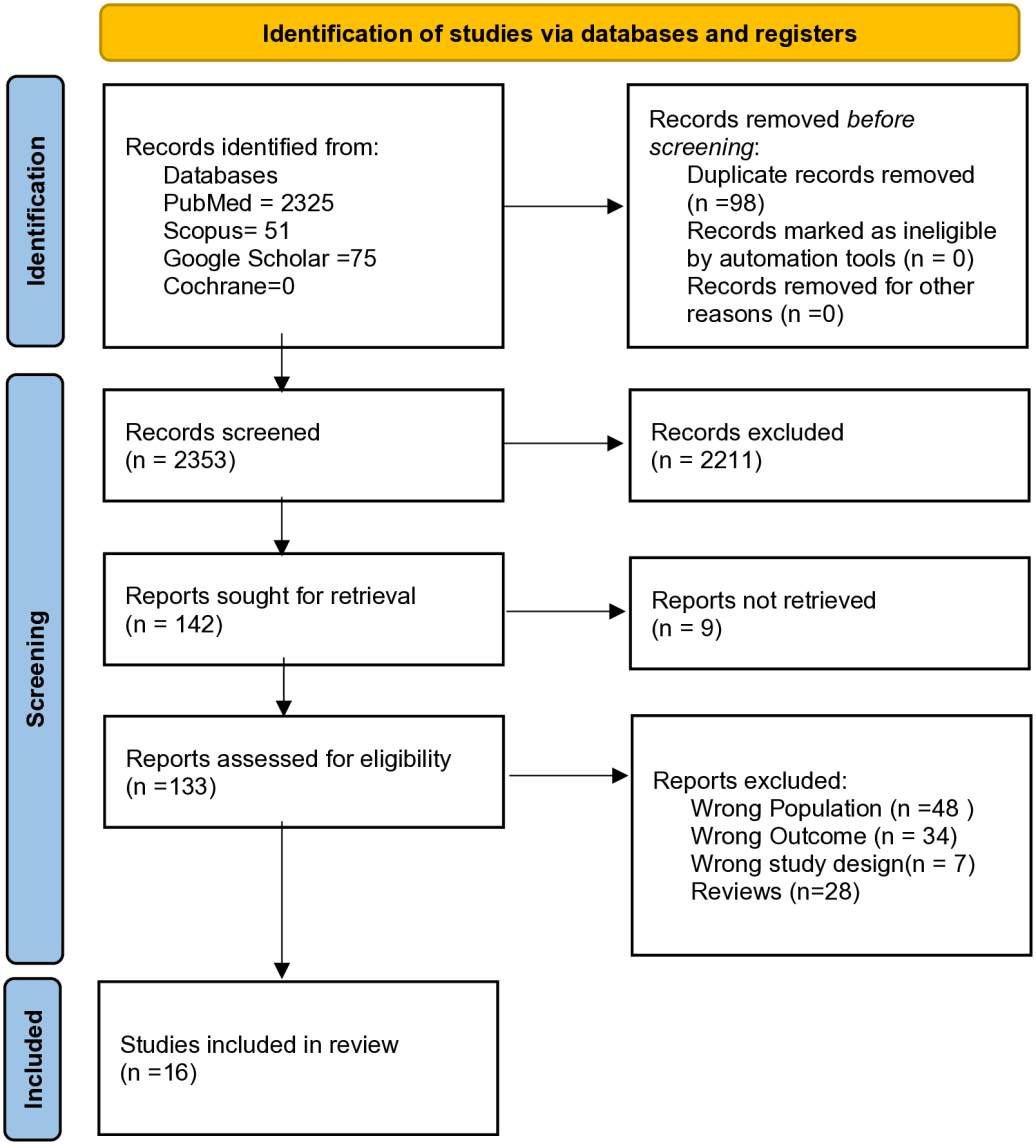
Studies selection process through PRISMA.

The search yielded 2451 studies, of which 2325 were identified from the PubMed, 51 from the Scopus, and 75 from the Google Scholar. Prior to the initial screening, 98 duplicates were removed. Subsequently, 2353 studies underwent title and abstract screening, of which 142 studies were included for comprehensive full-text screening. 133 studies were retrieved for full-text screening as we were unable to retrieve full-text on 9 studies due to unavailability of full-text. Of these, 28 review papers were excluded, along with 48 studies that did not align with the specified population criteria. Further, 34 studies were excluded as they did not have the specified outcome criteria and 7 studies did not meet the study design criteria. This resulted in inclusion of 16 studies [25–40] that met eligibility criteria for this review as shown in the Fig 1. The data was obtained from the 16 included studies.

Of the sixteen included studies in this review, four studies were cross-sectional [28,32,33,37], one study was observational [39], two studies were pre-post time-series [29,30], while two studies used mixed methods [25,36] and another study was qualitative [31]. Six studies did not report study design [26,27,34,35,38,40]. The included studies were published between 2018 and 2023. The included studies were mostly from the sub-Saharan Africa, South Asia, Southeast Asia and West Africa. The included countries were Tanzania, Zambia, Ethiopia, Kenya, Nigeria, Gambia, South Africa, Madagascar, Niger, Guinea, Pakistan, India, Vietnam, Ghana, and Mozambique. Most studies focused on operational aspects of vaccine management, including electronic immunization registry implementation (Vietnam, Tanzania, Zambia, Pakistan), vaccine wastage assessment (Ghana, Mozambique, Pakistan, Gambia), stock management and availability (Kenya, South Africa, India), and supply chain optimization (Guinea, Madagascar, Niger). The characteristics of these studies are provided in Table 1.

**Table 1:** Characteristics of included studies.

Title	Author	Year	Study Location	Study Design	Population
The Transition to an Entirely Digital Immunization Registry in Ha Noi Province and Son La Province, Vietnam: Readiness Assessment Study.	Duong H et al. [27]	2021	Ha Noi and Son La provinces in Vietnam	Not Reported	Health facilities and the NIIS end users in Ha Noi and Son La provinces in Vietnam.
Vaccine Cold Chain Management and Associated Factors in Public Health Facilities and District Health Offices of Wolaita Zone, Ethiopia.	Erassa et al. [28]	2023	Wolaita Zone, Ethiopia	Cross-sectional study	Health workers at EPI unit in public health facilities of Wolaita Zone
Vaccine wastage in Ghana, Mozambique, and Pakistan: An assessment of wastage rates for four vaccines and the context, causes, drivers, and knowledge, attitudes and practices for vaccine wastage.	Mvundura et al. [34]	2023	Ghana, Mozambique, Pakistan	Not Reported	District vaccine stores, sampled SDPs/facilities, regional/provincial warehouses, and national warehouses involved in vaccine supply chain
Added Value of Electronic Immunization Registries in Low- and Middle-Income Countries: Observational Case Study in Tanzania.	Secor et al. [37]	2022	Tanzania	Cross-sectional study	Individual-level patient visit dataset from the TImR electronic immunization registry system across multiple regions and health facilities in Tanzania.
Ensuring vaccine potency and availability: how evidence shaped Gavi’s Immunization Supply Chain Strategy.	Prosser et al. [36]	2022	Guinea, Kenya, Pakistan	Prospective mixed methods study design	Key informants at various levels of health systems in Guinea, Kenya and Pakistan
Using a low-cost, real-time electronic immunization registry in Pakistan to demonstrate utility of data for immunization programs and evidence-based decision making to achieve SDG-3: Insights from analysis of Big Data on vaccines	Siddiqi et al. [38]	2022	Sindh, Pakistan	Not Reported	Children enrolled in ZM-EIR
Redesigning immunization supply chains: Results from three country analyses.	Prosser et al. [35]	2021	Guinea, Madagascar Niger	Not Reported	Stakeholders from Ministries of Health and immunization programs
Factors that affect vaccines availability in public health facilities in Nairobi City County: a cross-sectional study.	Kanja et al. [33]	2021	Nairobi	Descriptive Cross-sectional	Public health facilities within Nairobi City County in Kenya, focusing on healthcare providers, administrators, and stakeholders involved in vaccine distribution and management.
Applying a governance barometer to vaccine delivery systems: Lessons from a rural district of Pakistan	Zaidi et al.[40]	2020	Pakistan	Not Reported	Healthcare providers, administrators, and stakeholders associated with vaccination programs
Using the consolidated framework for implementation research (CFIR) to assess the implementation context of a quality improvement program to reduce missed opportunities for vaccination in Kano, Nigeria: a mixed methods study	Adamu et al. [25]	2019	Nigeria	a formative cross-case evaluation with a convergent mixed methods design	Healthcare facilities and stakeholders involved in vaccination programs in Kano, Nigeria.
Vaccine wastage in The Gambia: a prospective observational study	Usuf et al. [39]	2018	Gambia	Observational Study	Healthcare facilities across multiple regions in Gambia involving vaccination programs administered to children and adults within these regions.
Mobile reporting of vaccine stock-levels in primary health care facilities in the Eastern Cape Province of South Africa: perceptions and experiences of health care workers	Iwu et al. [31]	2020	Eastern Cape Province, South Africa	Qualitative Research Design	Health care workers
The impact of an integrated electronic immunization registry and logistics management information system (EIR-eLMIS) on vaccine availability in three regions in Tanzania: A pre-post and time-series analysis	Gilbert et al. [29]	2019	Tanzania	Pre-post and time-series analysis	Health facilities
Vaccine stock management in primary health care facilities in OR Tambo District, Eastern Cape, South Africa	Iwu et al. [32]	2020	OR Tambo District, Eastern Cape, South Africa	Descriptive cross-sectional study	Nurses, pharmacists and pharmacist assistants who are employed on a full-time basis at each selected facility
Programmatic assessment of electronic Vaccine Intelligence Network (eVIN)	Gurnani et al. [30]	2020	India	Pre-post study design	Cold chain points
Design, Development, and Deployment of an Electronic Immunization Registry: Experiences From Vietnam, Tanzania, and Zambia	Carnahan et al. [26]	2023	Vietnam, Tanzania, Zambia	Not Reported	EIR implementation initiatives

### 3.1 Context setting

**Fig 2:**
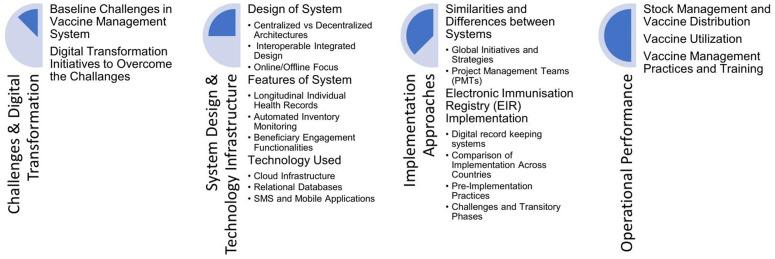
Result ramework.

The implementation of digital vaccine management systems in the LMICs has become a response to persistent challenges faced in maintaining effective cold chain management and ensuring vaccine availability. Despite diverse settings, countries faced common challenges of high rates of vaccine wastage due to improper handling practices, frequent stock outs at service delivery points, inadequate cold chain infrastructure, workforce capacity gaps, and weak data management systems that hindered evidence-based decision-making. These challenges in turn reduced immunization coverage and vaccine potency in resource-constrained environments with unreliable electricity, poor connectivity, and limited technical capacity. To overcome these challenges, countries adopted digital solutions ranging from Electronic Immunization Registries (EIRs) to comprehensive supply chain management systems to strengthen monitoring, improve stock visibility, reduce wastage, and enhance service delivery. The results section examines digital vaccine management systems in the LMICs across four dimensions: baseline challenges and digital transformation initiatives (section 3.1), system design and technology infrastructure (sections 3.2–3.4), implementation approaches and experiences across different country contexts (sections 3.5–3.6), and operational factors influencing vaccine delivery (section 3.7) as presented in Fig 2.

### 3.2 Cold chain management, challenges, and digital transformation across countries

Across the LMICs, assessments have shown persistent challenges in managing the cold chain for vaccine storage and distribution which is a major component of vaccine management system. In Ghana, Mozambique, and Pakistan, studies found high rates of health workers returning open vaccine vials to refrigerators instead of discarding them, as well as gaps in staff knowledge and defined stock management protocols, leading to concerning levels of vaccine wastage [34]. Similarly, factors such as supply chain issues, inadequate storage infrastructure, workforce shortages, and data management problems contributed to frequent vaccine stock-outs at public health facilities in Kenya [33]. To address such systemic gaps, some countries have made efforts to digitize their immunization data management and supply chain systems.

#### 3.2.1 Current cold chain management practices and digitalization.

**Vietnam’s NIS:** Vietnam has been transitioning from a paper-based to the digital National Immunisation Information System (NIIS), which enables the collection and consolidation of immunization coverage data, though the system still requires maintenance of both paper and digital records [27]. In 2010, Vietnam’s National Expanded Program on Immunization (NEPI) launched Project Optimize to boost immunization coverage. This initiative led to the creation of two digital systems: ImmReg for managing individual vaccination histories and VaxTrak for vaccine stock management. Initially, these systems were piloted at the district level. However, in 2011, lack of computers and internet connectivity at commune health centers prompted ImmReg and VaxTrak to be redeveloped as mobile applications for health workers. By 2013–2014, most commune health stations had computers and internet access, prompting the shift to a web-based application [26].

In 2017, ImmReg and VaxTrak were integrated into the National Immunization Information System (NIIS), significantly improving vaccination coverage and timeliness. By October 2022, NIIS included 32.2 million client records and was adopted by nearly all healthcare facilities nationwide. In late 2020, Vietnam initiated the transition to a paperless Electronic Immunization Record (EIR) system following a readiness assessment in two provinces, demonstrating its commitment to leveraging technology for healthcare improvement [26].

**Tanzania’s TImR and Zambia’s ZEIR:** Tanzania introduced the Tanzania Immunization Registry (TImR) in 2017. Though the initial system faced technical hurdles in 2014, TImR was launched as part of a broader package aimed at boosting data quality and use for immunization programs, which has been rolled out to over 3,700 facilities across 15 regions, providing insights on missed vaccination opportunities, population movements, and continuous quality improvement [26,37]. Similarly, in Zambia, Better Immunization Data Initiative (BID) led to the Zambia Electronic Immunization Registry (ZEIR) built on OpenSRP after an initial DHIS2-based attempt. Following a 2017 pilot, ZEIR reached 596 facilities across 29 districts by October 2022, capturing over 329,000 records, with plans for nationwide scale-up across 2,600 facilities. Zambia too involved user advisory inputs and local software partnerships for sustainability. ZEIR strengthened data quality, stock management, immunization monitoring, while offering digital child health cards for parents. Overall, the iterative, user-centred EIR designs in both nations catalysed enhancements in data reliability, supply visibility, and service delivery quality for immunization programs [26].

**Pakistan’s ZM-EIR:** The Zindagi Mehfooz (Safe Life; ZM) Electronic Immunization Registry (ZM-EIR), initially piloted in Karachi in 2012, was subsequently scaled up across the Sindh province of Pakistan between October 2017 and March 2018. By April 2019, the ZM-EIR was functional across 1539 facilities in 28 districts of Sindh, with over 2.9 million children and 0.9 million women enrolled, and more than 22 million immunization events recorded [38].

**India’s eVIN:** India has taken a more comprehensive approach, developing the Electronic Vaccine Intelligence Network (eVIN), a digital logistics management information system that has demonstrated improvements in vaccine utilization, stock management, and data quality across the immunization supply chain [30]. The introduction of eVIN in India has led to significant efficiency gains in the country’s vaccine distribution and management system. One of the key gains has been substantial reduction in vaccine wastage. After eVIN implementation, the utilization of vaccine doses decreased by 29.6%, from 305.3 million doses in the pre-eVIN period to 215.0 million doses post-eVIN, translating to savings of approximately 90 million doses due to reduction in vaccine wastage. This remarkable decrease in wastage can be attributed to improved visibility and monitoring across the supply chain, enabling better demand forecasting and distribution planning. Furthermore, eVIN has enhanced adherence to the ‘First Expiry, First Out’ (FEFO) principle, as evident by the reduction in the mean number of days until vaccine expiry from 428 to 384 at the cold chain point level. Improved record-keeping practices, with a 42.3% increase in the utilization of Government of India’s registers and a 93.8% reduction in the use of loose papers, have also contributed to greater efficiency. Additionally, the post-eVIN period witnessed a 57% decrease in the average replenishment time between vaccine indenting and supply across facilities, facilitating timely availability and mitigating stock-outs.

**Other countries:** Meanwhile, the system-level evaluations in Mozambique, Guinea, and Niger have identified opportunities to improve the efficiency and reliability of immunization supply chains through redesigned distribution networks and processes [35]. In Nigeria, the implementation of a quality improvement program to reduce missed vaccination opportunities was influenced by organizational factors like leadership, resources, and stakeholder engagement [25].

Lastly, an assessment in Ethiopia’s Wolaita zone revealed that while 61% of facilities demonstrated good cold chain practices, sustained improvements would require strengthening infrastructure, processes, and capacity building efforts [28]. Collectively, these countries examples highlight the multi-faceted nature of strengthening immunization supply chains and cold chain management, encompassing both digital innovations and targeted interventions to ensure reliable vaccine availability and potency. Details on vaccine management systems and processes of these countries are provided in Table 2.

**Table 2:** Vaccine management systems and processes in LMICs.

Country	System	Process
Vietnam	National Immunization Information System (NIIS) in 2017	Online immunization registries and tracks vaccine supply chain
		ID given to newborn within 24 hours of birth and managed throughout lifetime
		Used in computers, tablets or phones by healthcare workers
		Individual follow-up of vaccination status
		Tracking defaulters (people who are overdue for a vaccine dose);
		Automatically generating messages for recalls to alert people about overdue doses and for reminders to alert people about upcoming doses
		Provider assessment and feedback
		Stock management.
		Interoperability
South Africa	Stock visibility solution	Captures and monitors stock levels of vaccines using mobile phones to ensure continuous availability of vaccines
		HCWs send info about stocks to manager weekly and then manager has to respond to any facility with shortages by calling them and confirming whether the product has been ordered from depot or source it from other facilities with stock
Ethiopia	Cold chain and vaccine management system	Initiated cold chain monitoring systems
Tanzania	Electronic Immunisation Registries	Register, store and track immunization data
	Open Immunize (OpenIZ)	SMS reminder
	(TImR) Tanzania Immunization Registry	Stock management
	2017	Performance measurement reports
		Immunisation management
		Data compressed during synchronization so less internet bandwidth needed
		Works offline for weeks till data can be synced online
Zambia	Open SRP smart registry platform (ZEIR) in 2017	Tracks healthcare services to children under 5 years of age
		Unique identification
		Vaccination schedule
		Stock monitoring
		Creation of reports regarding the daily service data or monthly
Gambia	MyChild Solution	Relies on smart paper technology (filled at the point of care and scanned for automatic digitization) thus ensuring that vaccination can be done even in settings with limited infrastructure
	In 2017	Birth registration, record immunization received
		Vaccine stock management
		Automatic creation of reports
		Data integrated with DHIS-2
India	eVIN	Monitors temperature of cold chain through mobile phone and digitizes stocks
		Aids cold chain handlers by providing a complete overview of time of vaccine replenishment, supply and consumption patterns.
Pakistan	Zindagi Mehfooz (Safe Life) in 2018	SMS reminder
		Unique identification through quick-response barcodes linked to digital immunization records
		Web-based dashboard to generate immunization reports

Having established the challenges driving digital transformation and diverse responses and adaptations to these challenges, the following sections examines the technical design and features of digital systems implemented to address the challenges mentioned above.

### 3.3 Design of system

The design of a vaccine management system focuses on robustness, usability, and connectivity to address the challenging environment [41]. Our findings focus on the architecture, interoperable integration and the online and offline focus of the design of electronic vaccine management systems.

#### 3.3.1 Centralized vs decentralized architectures.

Countries like Vietnam, Tanzania and India invested in developing national-level centralized electronic registries to enable consolidated monitoring of vaccination coverage and supply chain indicators across provinces/districts by federal administrators [27,29,30]. Subsequently, South Africa’s Eastern Cape province piloted a targeted stock visibility system to serve inventory transparency needs between regional warehouses and facilities for context-specific insights [32].

#### 3.3.2 Interoperable integrated design.

Interoperability is the ability of a technical system to exchange and make use of information [42]. Vietnam’s National Immunisation Information System (NIIS) architecture exemplified interoperable capabilities allowing plug-and-play integration—a technology that allows peripheral devices to be connected to a computer and used almost immediately—with external health agencies databases for secure data exchange [27]. Similar intentions are visible in India which aimed to boost interoperability across the national eVIN network for immunization related data sharing between authorities [30]. Tanzania also embraced open Application Programming Interface(API)-based interconnection between their immunization registry and allied platforms hosting beneficiary health records for a holistic view [29].

#### 3.3.3 Online/offline focus.

While electronic registry systems in Tanzania and Zambia focused on harnessing cloud storage for online transaction processing on a large scale, Vietnam’s NIIS prioritized offline accessibility through local servers at facilities to enable uninterrupted recording by staff at rural centers with unreliable connectivity [27,29]. Along similar lines, Pakistan and South Africa’s Short Messaging Service(SMS)-based mobile application is designed to be tolerant to intermittent networks in remote areas for vaccine stock visibility [31,38].

### 3.4 Features of system

Features refer to the specific functions, capabilities, and technical attributes built into the software and hardware that allow the system to effectively manage the complexities of the vaccine supply chain [43,44]. This review identified 3 important features, namely, longitudinal individual health records, automated inventory monitoring, and beneficiary engagement functionalities.

#### 3.4.1 Longitudinal individual health records.

Systems like Vietnam’s NIIS, Tanzania’s Immunization Registry and Zambia’s ZEIR invested in functionalities for detailed recording of vaccination events at an individual child level [26,27,29]. This facilitated the monitoring of immunity levels across benefiting populations longitudinally over years spanning multiple locations. Tanzania’s system additionally allowed geospatial analytics mapping vaccination rates to sub-national trends.

#### 3.4.2 Automated inventory monitoring.

Core supply chain capabilities featured across all systems included vaccine stock tracking, expiry alerts, reordering notifications and wastage audits as visible in Tanzania’s architecture and Vietnam’s NIIS [27,29]. South Africa’s Stock Visibility System supplemented dashboard-based monitoring by configuring SMS triggers to notify administrators automatically in case of observed stock breaches against preset thresholds [31].

#### 3.4.3 Beneficiary engagement functionalities.

Zambia’s Electronic Immunization Registry design uniquely incorporated community-facing features like digital child health cards containing records accessible by parents using unique identification and SMS communication gateways to keep citizens informed about vaccination schedules [26]. This exemplified an intentional focus towards improving customer experience complementing provider-centric monitoring.

### 3.5 Technology used

#### 3.5.1 Cloud infrastructure.

Tanzania’s immunization registry and Zambia’s electronic system invested in cloud-based architectures built on platforms like District Health Information Software 2 (DHIS2) which enabled economical processing for analytical tasks [26,29]. Their shared infrastructure models allowed starting small while seamlessly scaling to regional and national levels progressively based on growing vaccination data demands. Zambia later switched to Zambian Electronic Immunization Registry (ZEIR) based on the Open Smart Register Platform (OpenSRP)—an open-source, mobile-based platform, built to enable data-driven decision making at all levels of the health system [26]. Similarly, Tanzania rolled out Tanzania Immunization Registry (TImR)in 2016, focussing on better data quality and data use for a smooth transition towards paperless registry [26,29].

#### 3.5.2 Relational databases.

Relational Structured Query Language (SQL) databases were specifically integrated across architecture layers in Vietnam’s NIIS design indicating the need for streamed data ingestion from hospitals and clinics at large volumes while also supporting complex analytical queries for long term policymaking [27].

#### 3.5.3 SMS and mobile applications.

The ubiquity of mobile devices and cellular networks across developing countries has led systems like South Africa’s stock visibility tool, and similar platforms in Zambia, India and Pakistan have adopted mobile/web applications and SMS gateways [26,30,31,38]. These supplemented dashboard-based access to widen accessibility for health workers and citizens by tapping into ubiquitous communication channels.

### 3.6 Similarities and differences between systems

#### 3.6.1 Implementation context and strategies.

The LMICs exhibit diverse management and operational approaches within their regions. Despite this diversity, there are both stark similarities and differences in the cold chain management systems across the LMICs. Understanding these distinctions is vital for comprehending success stories, best practices, and challenges in implementing cold chain management systems and Electronic Vaccine Management systems [36].

#### 3.6.2 Global initiatives and strategies.

At the global level, institutions like the Gavi and the World Health Organization have initiated similar programs following evidence based practices. The Global Immunisation Supply Chain (iSc) Strategy 2015–2020, launched in 2014, exemplifies such efforts. Subsequently, the Gavi introduced the Cold Chain Equipment Optimization Platform (CCEOP) in 2018, aimed at assisting countries in upgrading their cold chain systems by equipping them with reliable equipment and collaborating with private sector service providers. Evaluation of CCEOP in Guinea, Kenya, and Pakistan provided valuable insights into the adoption of this strategy and its operational success driven by its reliability, efficiency and low maintenance, making it sustainable. [26,40].

#### 3.6.3 Project management teams (PMTs).

As per the study by Prosser et al, Project Management Teams (PMTs) were created in the Ministries of Health of Guinea, Kenya, and Pakistan. These teams served as technical support and implementation bodies, overseeing equipment installation and collaborating with private sector partners [35]. The primary mission of PMTs was to ensure equitable vaccine distribution and deployment of technology to meet each country’s immunization needs. While PMTs were successful in various operational aspects, differences were observed, particularly in the evolution of PMT structures to the sub-national level in Kenya and Guinea, leading to ownership gaps and operational challenges at lower levels [36].

### 3.7 Electronic immunisation registry (EIR) implementation

#### 3.7.1 Comparison of implementation across countries.

The Electronic Immunisation Registry (EIR) plays a crucial role in collecting individual-level data, with increasing adoption in the LMICs. Evaluation of EIR implementation in Vietnam, Tanzania, and Zambia provided insights into prevalent systems and practices [26]. These countries initiated EIR incorporation around 2010, with Vietnam being the only country to fully scale up the program nationally within seven years. Key similarities across these countries included prioritizing national ownership and sustainability, with diverse national-level teams comprising leadership, technology, and immunization specialists.

#### 3.7.2 Pre-implementation practices.

Before EIR implementation, all three countries relied on paper-based forms for immunization-related activities such as stock management, reporting, and delivery. Additionally, interoperability considerations were addressed in all countries, with Vietnam creating an API based on HL7/FHIR for data sharing, Tanzania integrating TImR with VIMS, and Zambia transitioning ZEIR to integrate with DHIS2, in their national Health Information System [26].

#### 3.7.3 Challenges and transitory phases.

Studies conducted within these countries revealed challenges and transitory phases during the shift towards electronic systems. For instance, a study in two provinces of Vietnam highlighted the coexistence of paperless and paper-based systems under NIIS, along with the need for additional staff training to address entry mismatches [27].

### 3.8 Operational performance factors

#### 3.8.1 Stock management and vaccine distribution.

Missed Opportunities of Vaccination (MOV) are a concern in the LMICs, where stock outs are the primary reason for MOVs in countries with available data [37]. The management of stocks in the cold chain is essential for a continuous supply of vaccines. Shortages of cold chain equipment and vaccine supplies at vaccination points have been reported in several countries in the LMICs [28]. The reasons for this are unclear, but they reaffirm that there are many influencers of stock availability, including national-level stock outs and distribution systems, the capacity of staff to manage stock and submit orders, and the need to update forecasted demands with the extended reach of immunization services [32]. Some of the reasons for stock outs were the unavailability of the products at the vaccine depots and unreliable deliveries, vaccine stock status, management, and distribution, as well as the infrastructure supporting their availability [39].

Effective vaccine distribution at primary care settings and session sites relies on sound cold chain management. In several countries, outreach preparedness is hampered by the absence of micro-plans, insufficient fuel, and functional motorbikes. Weak supervision of routine immunizations, coupled with a lack of communication resources and social mobilization, further complicates the situation [40]. To ensure vaccine availability at session sites, improvements are needed in inventory management, timely deliveries, and related factors.

Vaccine potency is at risk during transportation due to freezing and heat exposure. Reducing travel duration outside the active Cold Chain minimizes these risks. Increasing distribution centers decreases reserve stock levels and holding costs while ensuring reliable, on-time vaccine delivery to session sites. This approach also helps maintain safe temperature conditions during transportation, benefiting from better conditioning processes. In resource-constrained developing countries, extending the distribution networks to the last tier should be coupled with identifying and acting on optimization opportunities.

#### 3.8.2 Vaccine utilization.

Ensuring effective cold chain management of vaccines is vital to vaccine utilization, particularly in the LMICs, where vaccine wastage is a common issue. The frequency of vaccination sessions is associated with wastage rates, suggesting that holding fewer sessions may reduce wastage by consolidating children and increasing the number of children in each session. Interestingly, facilities with fewer sessions might still experience higher wastage, possibly because of the smaller number of children in the sessions. Nationally, there is a need to improve vaccine wastage-related training, sensitization, and supportive supervision of the healthcare staff. Encouraging non-punitive reporting and monitoring can enhance transparency and trust, aligning stakeholders toward the common goal of decreasing vaccine wastage while maintaining and improving coverage. Reconsidering target wastage rates is also advisable, especially if they prove inconsistent across settings and strategies due to poor documentation or inadequate data or reporting, potentially impeding immunization goals [34].

#### 3.8.3 Vaccine management practices and training.

Effective vaccine management practices are vital for the proper handling of cold chains. Studies indicate that while cold chain handlers receive extensive training on vaccination and cold chain management, however, their training in inventory management is comparatively limited. Despite providing training to health workers and Cold Chain Equipment (CCE) technicians, the overall maintenance system remains inadequately strengthened. The insufficient maintenance of cold chains adversely affects vaccine efficacy due to frequent power outages and a lack of electricity backup [34,38]. Ensuring sustainability and system strengthening involves enhancing the reliability and efficiency of CCEs, thereby reducing the need for immediate and frequent maintenance [36].

#### 3.8.4 Documentation.

Documentation is another serious factor that affects cold chain management. Initially, for vaccine records and management, paper formats were used, such as a comprehensive log book for details of Cold Chain Equipment, stock management, repair and vaccine distribution, and a vaccine and logistics indent form for recording temperature, maintenance, and repair details of CCEs, for the vaccine stock, daily issues, and logistics indents. Recently, advancements have been made in shifting these to technological systems to address the gaps and strengthen cold-chain management [32]. The lack of documentation in maintenance poses serious challenges to the quality of recording, reporting, and monitoring of CCEs. And gaps were related to indifferent use of routine immunization data by health management, systems incomplete vaccination reports, and weak field supervision.

## 4 Discussion

This is the first comprehensive scoping review that aims to identify the characteristics of different vaccine management systems for routine immunization in the LMICs, factors affecting these systems, and the extent of digitization adopted to improve vaccine delivery. The findings from this review will help informing policy decisions and designing interventions to strengthen immunization supply chains and cold chain management practices across the LMICs. By systematically mapping diverse systems, challenges, and digital innovations implemented in different countries, this review presents a unique opportunity for cross-learning and adoption of best practices. The insights gained can guide policymakers and stakeholders in addressing systemic gaps, leveraging technological solutions to enhance vaccine availability, potency, and overall immunization coverage in resource-constrained settings and possibly allocating resources effectively. The studies elucidate several challenges and factors impacting vaccine management systems in these resource-constrained settings.

The design of cold chain management systems in the LMICs reveals both similarities and differences. Centralized and decentralized architectures focus on interoperability and offline accessibility, utilizing cloud infrastructure, relational databases, and mobile applications. Core system features include longitudinal health records, automated inventory tracking, supply chain management, and engagement functionalities including digital health cards. Implementation is influenced by global initiatives—Gavi’s Cold Chain Equipment Optimization Platform and local factors such as the evolution of sub-national oversight teams, transitional challenges of concurrent paper and electronic recordkeeping, and training needs.

The transition towards digitizing immunization data management and supply chain systems across the LMICs has exhibited both commonalities and distinct variations. Overarching global initiatives, such as Gavi’s immunization supply chain strategies and platforms, have provided common frameworks for countries to align their approaches. However, the system design choices have diverged based on contextual priorities – while some nations, including Vietnam, Tanzania, and India, have invested in centralized national-level electronic registries, and others, like South Africa, have piloted decentralized systems tailored to regional or facility-level stock visibility requirements for further scale-up. Architectural decisions have also differed, with some favouring cloud-based infrastructures for economical scaling, while others have prioritized offline operability through localized servers. Implementation factors have revealed further differences—the structure and evolution of project management teams overseeing deployments have varied across countries, influencing ownership and sub-national coordination. Timelines for comprehensive national scale-up of EIRs have also diverged, with Vietnam achieving full coverage within 7 years, while others are still working towards the scaling up of EIRs, coverage, or architecture. Moreover, transitory challenges, such as the concurrent use of paper-based and paperless systems during digitization, have underscored country-specific capacity gaps requiring localized training and process realignments. These contrasts highlight how global best practices have provided unifying strategies, yet bespoke design decisions and operational models have been necessitated by the distinct resources, capabilities, and health system characteristics of the LMICs.

Operational factors impacting performance include vaccine utilization and wastage minimization, stock management issues—shortages and unreliable forecasting, distribution weaknesses in outreach planning and transport resources, temperature exposure during prolonged vaccine transport, suboptimal maintenance due to power disruptions, and gaps in documentation practices.

A primary issue identified is the inadequate infrastructure and equipment necessary for maintaining the cold chain. This encompasses significant shortages of reliable cold rooms, freezers, and temperature monitoring devices, a cross-sectional study in India also reports similar challenges [45].

The absence of adequate storage of facilities heightens the risk of vaccine potency loss and wastage. Furthermore, persistent workforce shortages, training gaps, and a lack of defined protocols for essential practices, such as vaccine handling and stock management, exacerbate vaccine wastage and stock-outs. Similar challenges were identified in a study conducted in Ethiopia. These challenges were overcome by training of health workers and following appropriate guidelines along with longer work exposure to the process [46].

Another major challenge is frequent electricity outages and the absence of backup power sources, which further undermine cold chain integrity during vaccine transportation and storage. Long travel distances to distribution points also increases the duration that vaccines remain outside controlled temperature conditions, compromising their efficacy [47]. Additionally, inefficiencies in distribution networks, forecasting errors, and stock management problems at both national and sub-national levels contribute to downstream vaccine stock-outs at health facilities.

Strategically, global and national initiatives, such as the Global Immunization Supply Chain Strategy which is led by Gavi and supported by Immunization Agenda 2030, the Cold Chain Equipment Optimization Platform launched by Gavi in 2016, and dedicated Project Management Teams established by Ministries of health, play a crucial role [31,32,48]. These initiatives guide evidence-based interventions, equipment upgrades, distribution network redesigns, and ownership models that align with the country-specific contexts. Structured, evidence-based approaches and stakeholder collaborations are vital for successful implementation.

Overall, digital transformation journeys across LMICs vary, yet holistic programs targeting workforce training, cold chain infrastructure upgrades, distribution network optimization, and data-driven monitoring enabled by user-centered digital solutions show promise in strengthening immunization supply chains.

Despite the progress made, certain gaps in the literature remain. Few studies have conducted in-depth comparisons of system design choices and implementation factors between countries - Vietnam, Tanzania, and Zambia. Detailed data on wastage rates, stock out causes, distribution challenges, and maintenance gaps are limited, with scarce evidence from the countries like India, Kenya, and Guinea. Research has disproportionately focused on the introduction of electronic systems in Tanzania and Zambia, with limited evidence on long-term impacts and sustainability in Vietnam. Centralized architectures in Vietnam prioritize national oversight, while decentralized flexibility is observed in South Africa’s stock visibility system. Interoperability support varies based on existing health information architectures across Vietnam, Tanzania, and Zambia. Implementation patterns differ regarding the evolution of localized operational structures in Kenya and Guinea and transitional phase management in Vietnam.

Vaccine wastage is a systemic issue in the LMICs, but the root causes related to session planning, targeting policies, and monitoring practices remain unclear, this may not extend to other immunization processes as well [49]. While stock outs are attributed to distribution and inventory challenges in Tanzania and Zambia, their underlying drivers across national supply chains are not fully explored. Deficiencies in transport resources, microplanning, and immunization supervision persist in Pakistan and Kenya, despite being recognized as barriers to outreach.

### 4.1 Practice, policy and future implications

Based on our findings, we suggest several areas for implementation for countries that are yet to adopt a comprehensive vaccine management system. This includes strengthening data collection, optimizing supply chain planning, reinforcing cold chain infrastructure reliability, enhancing pandemic preparedness frameworks, and introducing electronic management systems where absent.

While electronic systems may facilitate implementation processes, their adoption needs an investment in training and capacity building of the workforce. Furthermore, the transition to electronic platforms requires substantial capacity building among existing human resources, representing a significant consideration for resource-constrained settings.

Further research is necessary to address these knowledge gaps and evaluate various aspects of vaccine management system design, implementation, and operations. Comparative assessments of the centralized system architecture used in Vietnam and the decentralized approach adopted in South Africa could reveal best practices suited to different contexts. Studying the interoperability strategies and change management techniques employed during Vietnam’s transition phase could provide insights for optimizing system rollouts. Investigations are needed to pinpoint the root causes of vaccine wastage, stock-outs, distribution bottlenecks, and maintenance issues in countries like Tanzania and Zambia, thereby informing evidence-based reforms.

Moreover, empirical studies are crucial to evaluate the impact of specific digital innovations and supply chain interventions on immunization coverage rates and cost-effectiveness measures. Implementation science methodologies can help identify the facilitators and barriers influencing the adoption of these interventions, deriving strategies to enable their sustainable scale-up across diverse settings in the LMICs. While global initiatives have increased equipment upgrades in countries like Guinea, Kenya, and Pakistan, sustained benefits require strengthened local operations and maintenance. Comprehensive training and supportive supervision in vaccine management, inventory control, documentation, and data utilization should be prioritized alongside technical system improvements in countries like Vietnam. Multisectoral collaboration involving governments, donors, technology partners, and communities can enhance holistic, context-adapted strategies for end-to-end cold chain optimization across LMICs.

### 4.2 Strengths and limitations

This scoping review fills an important knowledge gap by providing an overview of vaccine management systems, influencing factors, and the extent of digitization across the LMICs. The review adheres to rigorous methodological frameworks established by the Joanna Briggs Institute and PRISMA-ScR guidelines, ensuring a systematic and structured approach. Notably, the findings hold significant potential to inform and guide stakeholders in designing interventions and allocating resources judiciously to strengthen immunization supply chains and cold chain management practices, this is also corroborated by Pambudi et al [50]. However, certain limitations warrant consideration, such as including formal quality assessment of studies included due to the variation in study design. Additionally, the scope of this review is confined to routine immunization in the LMICs, which may limit the generalizability of findings to other contexts or immunization programs. Nevertheless, this scoping review represents an effort to synthesize the existing evidence and provide a comprehensive understanding of this critical domain

## 5 Conclusion

This review generates crucial evidence on various practices related to cold chain management system and delivery of vaccines across the LMICs. This review concludes that the LMICs have been adapting various systems including electronic system, digitization, decentralisation of vaccine delivery, stock monitoring, individual tracking to improve the delivery of vaccines. Across countries, some have made notable progress, while others are at their initial stage of implementation. Country level capacity differs largely becasue of inadequate training, technical knowlege, and digital infrastructure across health systems. Combining all aspects, this review provides ample scope to learn from each other. Although some countries are still at the initial stage of implementation, lessons learnt during this phase will guide scaling up initiatives. Despite differences in resource settings, difficulties faced while implementing electronic monitoring systems in India and Vietnam, can guide countries with paper-based systems. Moreover, rigorous scientific studies are required to gauge the impact of these initiatives and make inter-country comparisons.

## Supporting information

S1 File Search strategy. (DOCX)

S2 File PRISMA checklist. (DOCX)

## References

[1] Children: improving survival and well-being. https://www.who.int/news-room/fact-sheets/detail/children-reducing-mortality

[2] Immunization and Child Health. UNICEF India. https://www.unicef.org/india/what-we-do/immunization

[3] Vaccines and immunization. https://www.who.int/health-topics/vaccines-and-immunization

[4] WHO, UNICEF. Global childhood vaccination coverage holds steady, yet over 14 million infants remain unvaccinated. https://www.who.int/news/item/15-07-2025-global-childhood-vaccination-coverage-holds-steady-yet-over-14-million-infants-remain-unvaccinated-who-unicef

[5] Park K. Park’s Textbook of Preventive and Social Medicine. 27 ed. M/s Banarsidas Bhanot. 2023.

[6] Vaccines and immunization: What is vaccination?. https://www.who.int/news-room/questions-and-answers/item/vaccines-and-immunization-what-is-vaccination

[7] IA2030 draft 4. https://cdn.who.int/media/docs/default-source/immunization/strategy/ia2030/ia2030-draft-4-wha_b8850379-1fce-4847-bfd1-5d2c9d9e32f8.pdf?sfvrsn=5389656e_69&download=true

[8] World Health Organization. Guidelines for the international packaging and shipping of vaccines. World Health Organization. 2020. https://iris.who.int/handle/10665/338012

[9] Regulation and quality control of vaccines. https://www.who.int/teams/health-product-policy-and-standards/standards-and-specifications/vaccine-standardization/regulation-and-quality-control-of-vaccines

[10] Immunization handbook 107-198 part 2. https://www.who.int/docs/default-source/searo/india/publications/immunization-handbook-107-198-part2.pdf

[11] Supply chain. https://www.who.int/teams/immunization-vaccines-and-biologicals/essential-programme-on-immunization/supply-chain

[12] Evaluation of the Cold Chain Equipment Optimization Platform. 2025. https://www.gavi.org/our-impact/evaluation-studies/cceop-evaluation

[13] DSpace. https://iris.who.int/bitstream/handle/10665/44169/9789241563864_eng.pdf;sequence=1

[14] National Vaccine Wastage Assessment. https://www.unicef.org/india/media/6686/file/National%20Vaccine%20Wastage%20Assessment.pdf

[15] Setia S, Mainzer H, Washington ML, Coil G, Snyder R, Weniger BG. Frequency and causes of vaccine wastage. Vaccine. 2002;20(7–8):1148–56. doi: 10.1016/s0264-410x(01)00433-9 11803076

[16] Vaccine wastage rates and attributed factors in rural and urban areas in Uganda: Case of Mukono and Kalungu districts. PLOS Global Public Health. https://journals.plos.org/globalpublichealth/article?id=10.1371/journal.pgph.0003745 10.1371/journal.pgph.0003745 PMC12151339 40493663

[17] Vaccine Wastage Rates Calculator. https://www.who.int/publications/m/item/vaccine-wastage-rates-calculator

[18] Multi-dose vials versus single-dose vials for vaccination: perspectives from lower-middle income countries. https://www.tandfonline.com/doi/full/10.1080/21645515.2022.2059310 10.1080/21645515.2022.2059310 PMC9746400 35416750

[19] Next-generation immunization supply chains are needed to improve health outcomes. 2023.

[20] Improving cold chain systems: Challenges and solutions. https://www.sciencedirect.com/science/article/pii/S0264410X16307307 10.1016/j.vaccine.2016.08.045 27670076

[21] UNDP. Digital solutions for improved vaccine access. https://www.undp.org/asia-pacific/stories/digital-solutions-improved-vaccine-access

[22] World Bank country classifications by income level for 2024-2025. World Bank Blogs. https://blogs.worldbank.org/en/opendata/world-bank-country-classifications-by-income-level-for-2024-2025

[23] JBI Manual for Evidence Synthesis. JBI Global Wiki. https://jbi-global-wiki.refined.site/space/MANUAL

[24] PRISMA Extension for Scoping Reviews (PRISMA-ScR): Checklist and Explanation. https://pubmed.ncbi.nlm.nih.gov/30178033/10.7326/M18-0850 30178033

[25] Adamu AA, Uthman OA, Gadanya MA, Wiysonge CS. Using the consolidated framework for implementation research (CFIR) to assess the implementation context of a quality improvement program to reduce missed opportunities for vaccination in Kano, Nigeria: a mixed methods study. Hum Vaccin Immunother. 2020;16(2):465–75. doi: 10.1080/21645515.2019.1654798 31424313 PMC7062445

[26] Carnahan E, Nguyen L, Dao S, Bwakya M, Mtenga H, Duong H, et al. Design, development, and deployment of an electronic immunization registry: experiences from Vietnam, Tanzania, and Zambia. Glob Health Sci Pract. 2023;11(1):e2100804. doi: 10.9745/GHSP-D-21-00804 PMC9972371 36853635

[27] Duong H, Dao S, Dang H, Nguyen L, Ngo T, Nguyen T, et al. The Transition to an Entirely Digital Immunization Registry in Ha Noi Province and Son La Province, Vietnam: Readiness Assessment Study. JMIR Form Res. 2021;5(10):e28096. doi: 10.2196/28096 34694232 PMC8576599

[28] Erassa TE, Bachore BB, Faltamo WF, Molla S, Bogino EA. Vaccine cold chain management and associated factors in public health facilities and district health offices of Wolaita Zone, Ethiopia. J Multidiscip Healthc. 2023;16:75–84. 36660041 10.2147/JMDH.S385466 PMC9843497

[29] Gilbert SS, Bulula N, Yohana E, Thompson J, Beylerian E, Werner L. The impact of an integrated electronic immunization registry and logistics management information system (EIR-eLMIS) on vaccine availability in three regions in Tanzania: A pre-post and time-series analysis. Vaccine. 2020;38(3):562–9. doi: 10.1016/j.vaccine.2019.11.045 31706808 PMC6983926

[30] Gurnani V, Singh P, Haldar P, Aggarwal MK, Agrahari K, Kashyap S, et al. Programmatic assessment of electronic Vaccine Intelligence Network (eVIN). PLoS One. 2020;15(11):e0241369. doi: 10.1371/journal.pone.0241369 33151951 PMC7643996

[31] Iwu CJ, Ngcobo N, Cooper S, Mathebula L, Mangqalaza H, Magwaca A, et al. Mobile reporting of vaccine stock-levels in primary health care facilities in the Eastern Cape Province of South Africa: perceptions and experiences of health care workers. Hum Vaccin Immunother. 2020;16(8):1911–7. doi: 10.1080/21645515.2019.1700713 32096687 PMC7482903

[32] Iwu CJ, Ngcobo N, McCaul M, Mangqalaza H, Magwaca A, Chikte U. Vaccine stock management in primary health care facilities in OR Tambo District, Eastern Cape, South Africa. Vaccine. 2020;38(25):4111–8. 32362525 10.1016/j.vaccine.2020.04.019

[33] Kanja LW, Karimi PN, Maru SM, Kayumba PC, Hitimana R. Factors that affect vaccines availability in public health facilities in Nairobi City County: a cross-sectional study. Pan Afr Med J. 2021;38:72. 33889238 10.11604/pamj.2021.38.72.21580 PMC8033186

[34] Mvundura M, Ng J, Reynolds K, Theng Ng Y, Bawa J, Bambo M. Vaccine wastage in Ghana, Mozambique, and Pakistan: An assessment of wastage rates for four vaccines and the context, causes, drivers, and knowledge, attitudes and practices for vaccine wastage. Vaccine. 2023;41(28):4158–69. 37270365 10.1016/j.vaccine.2023.05.033

[35] Prosser W, Folorunso O, McCord J, Roche G, Tien M, Hatch B. Redesigning immunization supply chains: Results from three country analyses. Vaccine. 2021;39(16). 10.1016/j.vaccine.2021.03.037 33752952

[36] Prosser W, Sagar K, Seidel M, Alva S. Ensuring vaccine potency and availability: how evidence shaped Gavi’s Immunization Supply Chain Strategy. BMC Health Serv Res. 2022;22(1):1237. doi: 10.1186/s12913-022-08616-9 36207724 PMC9540167

[37] Secor AM, Mtenga H, Richard J, Bulula N, Ferriss E, Rathod M. Added Value of Electronic Immunization Registries in Low- and Middle-Income Countries: Observational Case Study in Tanzania. JMIR Public Health Surveill. 2022;8(1):e32455. doi: 10.2196/32455 PMC8817222 35060919

[38] Siddiqi DA, Abdullah S, Dharma VK, Shah MT, Akhter MA, Habib A, et al. Using a low-cost, real-time electronic immunization registry in Pakistan to demonstrate utility of data for immunization programs and evidence-based decision making to achieve SDG-3: Insights from analysis of Big Data on vaccines. Int J Med Inform. 2021;149:104413. doi: 10.1016/j.ijmedinf.2021.104413 33652259

[39] Usuf E, Mackenzie G, Ceesay L, Sowe D, Kampmann B, Roca A. Vaccine wastage in The Gambia: a prospective observational study. BMC Public Health. 2018;18(1):864. doi: 10.1186/s12889-018-5762-5 29996802 PMC6042329

[40] Zaidi S, Riaz A, Hussain SS, Omer SB, Ali A. Applying a governance barometer to vaccine delivery systems: Lessons from a rural district of Pakistan. Vaccine. 2020;38(3):627–34. doi: 10.1016/j.vaccine.2019.11.045 31699503

[41] Mobile Cold Chain Storage: Vaccine Cooling and Trasportations. Secop. 2024. https://www.secop.com/solutions/medical-cooling-solutions/vaccine-cooling-cold-chain-storage-and-solutions

[42] Sjarov M, Kißkalt D, Lechler T, Selmaier A, Franke J. Towards “Design for Interoperability” in the context of Systems Engineering. Procedia CIRP. 2021;96:145–50.

[43] Understand software features and their impact on development. https://www.harness.io/harness-devops-academy/mastering-software-features-tips-measurement

[44] Balzarini F, Frascella B, Oradini-Alacreu A, Gaetti G, Lopalco PL, Edelstein M, et al. Does the use of personal electronic health records increase vaccine uptake? A systematic review. Vaccine. 2020;38(38). 10.1016/j.vaccine.2020.05.083 32620374

[45] Kumar G, Gupta S. Assessment of cold chain equipments and their management in government health facilities in a District of Delhi: A cross-sectional descriptive study. Indian J Public Health. 2020;64(1):22–6. doi: 10.4103/ijph.IJPH_457_18 32189678

[46] Yassin. Knowledge of health professionals on cold chain management and associated factors in Ezha District, Gurage Zone, Ethiopia. Scientifica. 2019. 10.1155/2019/6937291 PMC6590539 31281711

[47] Dadari IK, Zgibor JC. How the use of vaccines outside the cold chain or in controlled temperature chain contributes to improving immunization coverage in low- and middle-income countries (LMICs): A scoping review of the literature. J Glob Health. 2021;11:04004. 33692889 10.7189/jogh.11.04004 PMC7915947

[48] Evaluation of the Cold Chain Equipment Optimization Platform. 2025. https://www.gavi.org/our-impact/evaluation-studies/cceop-evaluation

[49] Lazarus JV, Karim SSA, Selm L van, Doran J, Batista C, Amor YB, et al. COVID-19 vaccine wastage in the midst of vaccine inequity: causes, types and practical steps. BMJ Global Health. 2022;7(4). 10.1136/bmjgh-2022-009010 PMC9044511 35474267

[50] Pambudi NA, Sarifudin A, Gandidi IM, Romadhon R. Vaccine cold chain management and cold storage technology to address the challenges of vaccination programs. Energy Reports. 2022;8:955–72. doi: 10.1016/j.egyr.2021.12.039

