# Supplementary material for: Vaccine management systems with a focus on cold chain management and delivery of immunization services in low- and middle-income countries: A scoping review
Source: PLoS One. 2026 Jun 16;21(6):e0350231. doi: 10.1371/journal.pone.0350231 (PMC13271511; doi:10.1371/journal.pone.0350231)
Supplement: S1 File — (DOCX) [file pone.0350231.s001.docx]

**Appendix 2: Search Strategy**

**Scopus**

(ALL("cold chain management") OR ALL("vaccine utilisation") OR ALL("vaccine management") OR ALL("vaccine distribution") OR TITLE-ABS-KEY("vaccine delivery") OR ALL("vaccine wastage") OR ALL(vaccine transportation) OR ALL("vaccine storage") OR ALL("routine immunization") AND ALL("low middle income countries") AND NOT ALL(COVID))
